# Marine Microalgae–Microorganism Co-Cultures: An Insight into *Nannochloropsis* sp. Use and Biotechnological Applications

**DOI:** 10.3390/foods14091522

**Published:** 2025-04-26

**Authors:** Marta Vala Esteves, Diana M. C. Marques, Joana D. de Almeida, Nuno Torres Faria, Frederico Castelo Ferreira

**Affiliations:** 1 Department of Bioengineering and Institute for Bioengineering and Biosciences, Instituto Superior Técnico, Universidade de Lisboa, Av. Rovisco Pais, 1049-001 Lisbon, Portugal; m.vala.esteves@tecnico.ulisboa.pt (M.V.E.); diana.c.marques@tecnico.ulisboa.pt (D.M.C.M.); joana.d.almeida@tecnico.ulisboa.pt (J.D.d.A.); 2 Associate Laboratory i4HB—Institute for Health and Bioeconomy, Instituto Superior Técnico, Universidade de Lisboa, Av. Rovisco Pais, 1049-001 Lisbon, Portugal

**Keywords:** *Nannochloropsis*, co-culture, marine oleaginous microalgae, biotechnology, food, bioactive compounds

## Abstract

The increasing demand for sustainable, economical, and environmentally friendly solutions has positioned microalgae as promising candidates in biotechnology, particularly in food, feed, nutraceutical, pharmaceutical, biofuel, and bioremediation applications. This review explores the role of the *Nannochloropsis* genus and other marine oleaginous microalgae in co-cultivation systems, highlighting their mechanisms of interaction with various microorganisms and their potential for various biotechnological purposes. Case studies of *Nannochloropsis* sp. co-cultures with other microalgae, bacteria, and fungi are presented. The different types of associations are described as alternative strategies to enhance biomass productivity, lipid accumulation, and nutrient recycling. A key focus of this review is the potential of *Nannochloropsis* microalgae co-cultivation in food, as it is part of the list of microalgae to be approved for consumption in the European Union, discussing their rich nutritional value, safety, and regulatory status. Additionally, the role of microalgae in the alternative protein sector is explored, with particular emphasis on their integration in cultivated meat products as nutrient suppliers and metabolic partners for animal cells. Despite their potential, several challenges, such as scale-up, contamination risk, and strain selection, remain key obstacles to the widespread adoption of microalgal biotechnology. Future research should focus on optimizing microalgae-based co-cultures for food applications, addressing safety concerns, and further investigating their integration into functional foods and cellular agriculture products.

## 1. Introduction

Microalgae constitute a wide group of photosynthetic organisms that have proven their value in biotechnology. Their simple cultivation requirements, rapid growth rates, high photon conversion efficiency, and rich biochemical composition have brought significant attention to their use in a myriad of applications. Among the vast diversity of microalgae, marine oleaginous species have gained increasing attention for their potential in biotechnology. These microorganisms are highly valued for their ability to thrive under varying salinity and temperature conditions while biosynthesizing a wide array of secondary metabolites with value in food, biofuel production, and nutraceuticals [1]. Their high productivity in vitamins, proteins, pigments, minerals, and other bioactive compounds, combined with their rapid proliferation and simple growth requirements, makes them promising candidates for food and nutraceutical applications [2].

In fact, microalgae are highly prized in food applications due to their rich protein content, which can vary from 6 to 70% of their dry weight (DW), with a well-balanced amino acid profile that includes the essential amino acids required for human nutrition [3]. Moreover, microalgae present high rentability due to their high protein yield per unit of biomass, making them an efficient and cost-effective alternative for large-scale protein production [3]. In addition to their high content, microalgae-derived proteins have also been associated with health benefits, including antioxidant, anti-inflammatory, and antiproliferative effects, further increasing their appeal in the development of functional foods [4]. That said, marine microalgae have been used as a valuable source of high-quality proteins, offering a sustainable and nutrient-rich alternative to traditional protein sources.

Carotenoids of microalgae origin, such as β-carotene, astaxanthin, lycopene, and fucoxanthin, among others, hold significant market value due to their vibrant pigmentation and well-documented medicinal properties, making them highly relevant as natural colorants and functional ingredients in food formulations [2]. These compounds contribute to a healthy immune system and have been associated with the prevention of cardiovascular diseases, macular degeneration, and inflammatory conditions, as well as exhibiting antioxidant and antiproliferative activities [2].

In addition, marine microalgae represent a sustainable alternative to fish oils as a source of essential polyunsaturated fatty acids (PUFAs), particularly eicosapentaenoic acid (EPA) and docosahexaenoic acid (DHA) [1]. These omega-3 fatty acids (FAs) play a crucial role in cardiovascular health, inflammation regulation, and neurological function, with therapeutic applications in conditions such as Parkinson’s, Alzheimer’s, psoriasis, cancer, and rheumatoid arthritis [2]. While EPA serves as a precursor for bioactive lipids essential for cell membrane integrity, DHA is a major structural component of the brain, retina, and heart, underscoring its importance in human development and health [5]. Altogether, microalgae omega-3 and -6 FAs are of extreme value in human and animal nutrition, being highly prized in food and feed applications, especially given the incapacity of animals, including humans, to synthesize these compounds naturally, emphasizing the need to obtain them from dietary sources [6].

As the demand for natural, sustainable, and economically viable nutritional supplements continues to rise, optimizing microalgae bioprocesses for enhanced metabolite production remains a priority to ensure cost-effective and eco-friendly commercial applications. In this review, the role of microalgae–microorganism co-culture is displayed in the context of maximizing production pathways that bring advantages to various biotechnological fields, with a focus on studies conducted on particular marine microalgae species of great biotechnological interest. Table 1 summarizes the key bioactive compounds, lipid content, and applications of the species of marine oleaginous microalgae with increased biological interest.

From this list, *Nannochloropsis* stands out as a genus of particular interest due to its unique metabolic profile and broad biotechnological potential. Given the growing interest in co-cultivation as a strategy to develop more sustainable and efficient bioprocesses, along with the increasing focus on *Nannochloropsis*, it is important to consolidate the existing research on the topic. Its rich nutritional composition, coupled with its adaptability as a versatile co-culture partner, has positioned *Nannochloropsis* as a promising candidate for novel bioprocessing strategies. A comprehensive understanding of past and ongoing studies will help clarify the principles and advantages of *Nannochloropsis* co-culture with other microorganisms, ultimately paving the way for unlocking its full biotechnological potential.

*Nannochloropsis* spp. are marine oleaginous microalgae that belong to the Eustigmatophyceae class. The *Nannochloropsis* genus includes five well-characterized species: *N. oceanica*, *N. australis*, *N. granulata*, *N. oculata*, and *N. limnetica* (the only freshwater species) [19]. *Microchloropsis gaditana* and *Microchloropsis salina*, initially classified as *Nannochloropsis*, were reclassified to the *Microchloropsis* genus due to significant genomic differences [20]. For simplification, this review considers both *Microchloropsis* species as part of the *Nannochloropsis* group, as they share similar biochemical and nutritional properties.

*Nannochloropsis* species are single-celled, oval-shaped, non-flagellated organisms with dimensions typically in the range of 2 to 8 µm. These microalgae are characterized by their thick, rigid cellulose-rich wall, composed mainly of glucose (~68%) and, in minor concentrations (4 to 8%), other monosaccharides such as rhamnose, mannose, ribose, xylose, fucose, and galactose [21]. Scholtz et al. (2014) [22] conducted a detailed study on the cell wall structure and composition of *N. gaditana*, revealing a two-layered cell wall. The inner layer is predominantly composed of cellulose (~75%) while the outer layer is rich in algaenan, a highly hydrophobic biopolymer of long-chain hydrocarbons [22]. The authors proposed that this outer algaenan wall is a major contributor to the recalcitrance of *N. gaditana* cell walls, making them more resistant to enzymatic and chemical degradation [22]. Whilst this cell structure improves the organism’s ability to withstand harsh environmental conditions, it also presents a challenge in extracting intracellular compounds in industrial processes.

Unlike many other microalgae, *Nannochloropsis* cells are distinguished by their composition of chlorophyll a but not chlorophyll b or c [7]. Apart from the freshwater microalgae *N. limnetica*, *Nannochloropsis* species thrive in marine environments and are, therefore, cultured in artificial seawater-like environments. These species are grown under aerobic conditions in the presence of a nitrogen source and light radiation to support their photosynthetic regime. Nonetheless, some can adopt heterotrophic or mixotrophic regimes when supplied with organic carbon sources. Commercial cultivation is often carried out in open raceway ponds or closed photobioreactors under controlled optimal conditions to enhance productivity and metabolic performance [7].

The emerging focus on *Nannochloropsis* spp. is justified by their robust growth characteristics, ability to thrive in varying environmental conditions, and feasibility of large-scale cultivation, making them ideal candidates for sustainable biomass production. Additionally, they have a notable biochemical composition, mainly regarding their rich lipid content, representing more than 20% of their DW and up to 60% when cultured under stress conditions [23]. As oleaginous microalgae, *Nannochloropsis* sp. have a unique fatty acid profile, with the ability to synthesize a variety of saturated (SFAs), monounsaturated (MUFAs), and polyunsaturated fatty acids (PUFAs), the latter representing a significant share of the total fatty acid content [23]. In fact, the high PUFA content makes these green microalgae particularly valuable, especially for omega-3 dietary supplements and biofuel production. Their ability to synthesize significant amounts of EPA, which can constitute 2% or more of their dry weight, further enhances their biotechnological and nutritional appeal for all the associated health benefits. Additionally, the protein and carbohydrate content represent around 25–30% and 30% of their DW, respectively, enriching the nutritional value of *Nannochloropsis*. Nevertheless, these data vary across strains, culture conditions, methodology, and other factors. Moreover, the carotenoid profile is also noteworthy, namely, for the production of violaxanthin and, in minor concentrations, astaxanthin, canthaxanthin, zeaxanthin, and β-carotene, which also carry medicinal value, as previously stated [24]. As demonstrated, the nutritional value of *Nannochloropsis* is substantial and should be fully harnessed to enhance its applications in developing nutritious food, paving the way for more sustainable and health-promoting solutions. Recent advances in the biotechnology field have explored co-cultivation principles as strategies to enhance the sustainability, productivity, and cost-efficiency of microalgal bioprocesses, representing an advantage over mono-cultures [25]. Co-cultivation refers to growing two or more different populations of cells or whole microorganisms in a shared and controlled environment. The goal is to mimic the natural habitat of cells and stimulate their natural biotic and abiotic interactions, ultimately optimizing their bioactivity [26]. This approach leverages several ecological principles that naturally occur; for instance, different species that require complementary resources are able to co-exist together, with minimal competition and enhanced overall productivity [27]. The sustainable side of co-culture is directly related to the circular exchange of nutrients that occurs between autotrophic and heterotrophic species, mainly in recycling nitrogen and carbon compounds, closing the cycles [28]. In this way, the reciprocal exchange of nutrients between co-cultured species allows for a more efficient utilization of available resources, improving growth rates and metabolic output. Overall, there are several studies that show that co-cultivation experiments improve production rates and growth profiles and may trigger new production pathways, which increases chemical diversity [29].

This review focuses on the integration of marine oleaginous microalgae in co-culture systems with other microorganisms, which holds great interest in biotechnology with promising results in nutritional supplements, aquaculture, biodiesel production, and wastewater treatment (WWT) [25]. Their ability to engage in productive interactions with other microorganisms opens new possibilities for enhancing biomass productivity, improving nutrient recycling, and creating more sustainable bioprocesses. Given these advantages, reviewing the potential of *Nannochloropsis* in co-cultivation systems is crucial for advancing its applications in biotechnology and ensuring its full potential.

This review aims to explore the existing studies on the co-culture of marine oleaginous microalgae with other microorganisms, with particular focus on the *Nannochloropsis* species. It uncovers the mechanisms behind these interactions, identifies the most common biotechnological applications, and discusses the potential applications of *Nannochloropsis* co-cultures in the food industry. Finally, it presents the challenges and opportunities for future developments in marine oleaginous microalgae processes.

## 2. Materials and Methods

The Google Scholar and Clarivate Web of Science platforms were used to search for articles using the words “co-culture”, “marine microalgae”, “*Nannochloropsis*”, or “oleaginous” as mandatory. Other words, such as “biotechnology”, “biofuel”, “wastewater”, “food”, “feed”, “nutraceuticals”, or “cellular agriculture”, were defined as optional. The search was limited to the years between 2001 and 2024. From the retrieved articles, a careful selection process was applied to prioritize original research over review papers whenever possible.

## 3. Mechanisms of Interaction in Microbial Consortia

Over time, organisms have developed diverse strategies to interact with each other to better adapt to their environment, tolerate adverse conditions, and even resist invasive species. These interactions can take different forms, depending on the benefits or disadvantages conferred to the organisms in association. That said, symbiosis, the interaction between two organisms of different species, can be of different types: mutualism, when both parties benefit from the interaction; commensalism, when only one species is benefited while the other is unaffected; and parasitism, when one species gains an advantage at the expense of the other [30].

These associations are frequent in microbial communities, including microalgae–microorganism consortia. Compared to axenic cultures, co-cultivation is a strategy often applied as it results in enhanced productivity, microbial resilience, and metabolic efficiency, making poly-cultures extremely valuable in the bioprocessing field [25]. However, one of the main challenges in artificial poly-cultures is selecting the appropriate species before promoting naturally occurring interactions for industrial purposes. A thorough study on the impact of both biotic and abiotic factors on a co-culture system is essential to ensure that the selected strains are beneficial for industrial applications.

Robust microbial strains with high growth rates, strong tolerance to environmental fluctuations, and valuable metabolic activities are essential for cost-efficient and scalable bioprocesses. When designing a co-culture system for biotechnological large-scale applications, strain selection must consider not only the inherent biological value of the organisms but also their compatibility with industrial settings. For instance, given that *Nannochloropsis* is usually cultured under high salinity media, it is imperative to choose co-culture partners that exhibit halotolerance or halophilic traits. In fact, these characteristics are advantageous, not only because high salinity can suppress pathogenic species, mitigating contamination issues, but also because halotolerant microbes often offer metabolic versatility [31]. An example with industrial interest is the bacteria *Paracoccus* spp., known for their flexible metabolism, ability to survive on diverse substrates, effective denitrification capacity, and overall resilience to environmental changes [32]. Such traits could help optimize nutrient cycling and enhance lipid production in a *Nannochloropsis* culture, with prospects for scaling up. Therefore, a detailed evaluation of the environmental adaptability of microorganisms is important in strain selection for co-cultivation purposes. Ultimately, the strategic selection of robust, high-performing strains lays the grounds for scalable, cost-effective, and sustainable microalgal bioprocesses.

Another relevant aspect to account for in strain selection is the microbial mechanisms of interspecies interactions. Most microalgal interactions with other species occur in aquatic habitats through the exchange of metabolites or signaling molecules in the phycosphere—the rich microenvironment immediately surrounding a microalgal cell, crowded with various microorganisms [33]. The network of metabolites that is established in a microbial consortium can be categorized into two different mechanisms of communication based on the nature of the compounds involved, whether they are produced within cells (endometabolites) or secreted into the surrounding environment (exometabolites) [34].

### 3.1. Metabolite Exchange

One of the most fundamental interactions in microalgae–microorganism consortia is built on the exchange of nutrients. As photoautotrophic organisms, microalgae play a crucial role in the carbon cycle by fixing atmospheric carbon dioxide (CO_2_) and producing organic carbon substrates, essential micronutrients, and oxygen (O_2_). These metabolites serve as energy sources for the heterotrophic microorganisms in association, which, in return, release CO_2_ and inorganic carbon byproducts back into the environment to be used by microalgae, establishing a mutualistic carbon–oxygen cycle [34].

A similar exchange occurs with nitrogen compounds. Some nitrogen-fixing bacteria (e.g., *Azospirillum*, *Rhizobium*) can convert atmospheric nitrogen (N_2_) into bioavailable inorganic nitrogen, in the form of ammonium (NH_4_^+^) or nitrate (NO_3_^−^), ready to be utilized by microalgae [35]. The exchange of nutrients is also extended to sulfur compounds, derived from microalgal metabolism and subsequently utilized by sulfur-metabolizing bacteria and fungi, establishing another cycle within microbial communities [35].

### 3.2. Chemical Interactions

Beyond nutrient exchange, microalgae can interact with surrounding microorganisms through chemical signaling, which can trigger physiological responses and influence population dynamics by controlling gene expression. In microalgae, these interactions are mediated by allelochemical metabolites, a diverse group of signaling molecules that include pheromones, free fatty acids, alkaloids, aldehydes, peptides, or extracellular metabolites, among others [36]. Such exchange of metabolites can have either a positive or negative effect on neighboring communities: in some cases, microalgal allelochemicals inhibit the growth of competing species, disrupt biofilm formation, or induce cell lysis, working as antimicrobial agents [36]. In others, the byproducts of algal metabolism can induce an adaptive response, helping them and the surrounding organisms to better adapt to extreme environments or resist stress conditions, such as nutrient deprivation [37].

One of the most well-documented bacterial–chemical mechanisms of interaction, quorum sensing (QS), has been shown to influence microalgal behavior, affecting nutrient acquisition, biofilm formation, and self-motility [38]. In response, these photoautotrophic organisms have developed defense mechanisms, being able to produce QS inhibitors that interfere with bacterial communication systems [38]. The production of indole-3-acetic acid (IAA) is a well-studied example of a chemical signaling mechanism between microalgae and bacteria. When secreted into the extracellular space by *Sulfitobacter* species, the hormone IAA promotes microalgae growth and lipid production, which carries biotechnological value. However, at high concentrations, it becomes toxic for microalgae, favoring the bacteria that take advantage of the nutrients from the lysed cells [39,40]. This example underlies the importance of carefully studying microbial interactions since a beneficial relationship can rapidly shift into a competitive or even parasitic association.

Despite these interrelations depending highly on the metabolome of species, abiotic factors also play a part in allelopathy behaviors. Nutrient limitation, low temperatures, and high pH are known to stimulate the synthesis of allelochemicals, whereas high light irradiation, high temperatures, high nutrient bioavailability, and low pH repress the secretion of signaling molecules [36].

### 3.3. Metabolomics of Nannochloropsis

In *Nannochloropsis*, the biosynthesis of triacylglycerols (TAGs) and EPA involves a series of enzymatic reactions that convert simple FAs precursors into complex lipid molecules [7]. Summarily, TAG synthesis primarily occurs through the acyl-CoA-dependent Kennedy pathway, which comprises sequential acylation steps of glycerol-3-phosphate into lysophosphatidic acid, phosphatidic acid, diacylglycerol, and, finally, TAG by the action of various enzymes [41]. Under nitrogen-limited conditions, there is higher accumulation of TAGs, particularly C16 fatty acids, due to the overexpression of diacylglycerol acyltransferase genes [41]. Additionally, an acyl-CoA-independent pathway can also contribute to TAG production, especially in CO_2_-rich environments, by utilizing photosynthetically assimilated carbon [42]. Regarding EPA production, *Nannochloropsis* follows one metabolic route, i.e., the ω-6 pathway, which varies from other EPA-producing microalgae, such as *P. tricornutum*, that may employ two different pathways for EPA production [43]. In *Nannochloropsis*, EPA biosynthesis occurs in the endoplasmic reticulum, where stearic acid (C18:0) is converted into EPA (C20:5) in various intermediate steps through the action of fatty acid desaturates and a fatty acid elongase [42]. The newly synthesized EPA molecule is then transferred to the chloroplast membrane, a translocation process that constitutes a difficulty in cellular engineering approaches for increasing EPA production [42]. This unique, single-route pathway for EPA production not only highlights the species’ metabolic specialization but also points to both opportunities and challenges for future metabolic engineering aimed at enhancing EPA yields in industrial applications [7].

A thorough understanding of the lipid metabolic pathways in *Nannochloropsis* is crucial for selecting appropriate co-cultivation strains and optimizing culture conditions to maximize bioactive lipid production. As previously discussed, multiple factors influence the lipid metabolism of microalgae, including the presence of other microorganisms in culture. For instance, TAG accumulation can be stimulated in the presence of denitrifying bacteria that would contribute to the depletion of nitrogen in the medium [44]. Conversely, co-cultivation with photosynthetic microorganisms can promote competition for atmospheric carbon, potentially reducing *Nannochloropsis* metabolic activity and, therefore, lowering lipid productivity [44]. On the contrary, anaerobic bacteria capable of recycling organic carbon produced by microalgae, re-introduce it into the system, increasing the availability of photosynthetic-assimilated carbon and promoting lipid synthesis. Additionally, microalgae from the *Nannochloropsis* genus use B vitamins as growth factors and health promoters, but they lack the ability to synthesize them independently, needing an exogenous source [45]. Certain microorganisms can serve as a source of B vitamin precursors, enhancing biomass growth and indirectly stimulating lipid accumulation by boosting photosynthetic activity [45]. Hence, the presence of other microorganisms in co-culture systems can be a valuable asset for the enhanced biomass and lipid production of *Nannochloropsis*, as various microbes can secrete signaling molecules, enzymes, or metabolites that directly impact metabolic pathways.

Abiotic factors also play a fundamental role in microalgae metabolism and must be considered when designing co-culture systems. *Nannochloropsis* thrives at an optimal temperature of 25–29 °C, at a pH of 7–10, at salinity levels between 22 and 49 g/L, and in the presence of light as an obligate phototrophic species. Despite being species that can easily adapt to environmental fluctuations, stress conditions can have implications in the metabolic pathways of *Nannochloropsis*. At lower temperatures (down to 9 °C), the production of unsaturated lipids is enhanced as a mechanism to maintain membrane fluidity, which results in higher EPA and PUFA contents, while higher temperatures (up to 31 °C) promote the synthesis of TAGs [46]. While *Nannochloropsis* sp. are considered to be halophilic species, tolerating a wide salinity range, the salt concentration influences their growth and lipid profiles [47]. Studies indicate that at lower salinity levels (25‰), EPA accumulation increases while total lipid content decreases [47]. The content of C16 is not significantly altered, although it reaches higher levels in low salinity environments [47]. Light intensity and irradiation wavelength have also been reported to influence biomass growth and metabolite production in *Nannochloropsis*, with the total lipid content increasing with light exposure along with the levels of TAGs. However, this condition results in reduced EPA synthesis due to alterations in the chloroplast membrane, where EPA is allocated [48]. Moreover, the fatty acid profile is also responsive to variations in light wavelength, with blue and red light favoring the production of SFAs over white light [49].

All things considered, considering the influence of abiotic factors in *Nannochloropsis* cultures is fundamental when designing poly-culture systems. The previously presented studies show that fluctuations from standard cultivation conditions have an impact on *Nannochloropsis* growth and bioactivity. Hence, it is important to not only guarantee that the cultivation conditions are favorable for all involved species, ensuring optimum growth, but also to account for the influence that culture partners may have on the environmental settings. For instance, the high cell density of co-cultured partners can compromise light penetration, altering the culture conditions and influencing *Nannochloropsis* metabolic activity.

In fact, previous studies have demonstrated that co-cultivation strategies can impact the metabolism of microalgae through their mechanisms of interaction. Xu et al. (2018) [50] studied the co-culture of *Chlamydomonas reinhardtii* with *Azotobacter chroococcumg*, reporting that the co-cultivation strategy promoted the growth and lipid production of microalgae under nitrogen deprivation by upregulating microalgae genes responsible for lipid metabolism [50]. In a different study, Leyva et al. (2015) [51] concluded that the presence of *Azospirillum brasilense* in *Chlorella vulgaris* cultures promoted the activity of acetyl-CoA carboxylase and thereby enhanced lipid accumulation. Given that this enzyme is also part of *Nannochloropsis* lipid biosynthesis [7], co-culture strategies leveraging microbial interactions to enhance enzymatic activity could be explored while maintaining optimal cultivation conditions.

Further studies should focus on deepening the knowledge of the specific metabolic changes in *Nannochloropsis* when co-cultured with different microorganisms. Understanding the underlying regulatory mechanisms of lipid and biomass production will enable the selection of microbial partners that optimize microalgal productivity. Additionally, assessing how both biotic and abiotic factors impact co-culture systems will be essential for developing sustainable and efficient lipid production strategies.

The communication between microalgae and microorganisms is a highly complex and dynamic network of signaling molecules and metabolic exchanges, which can be either advantageous or harmful for the species involved. Hence, it is essential to conduct a strict study before inoculating two different species to ensure that the desired goals are achieved. Understanding the microbial ways of communication, the mechanisms of microalgae when in the presence of other organisms, and the competitiveness for resources is vital when designing a synthetic co-culture of microorganisms.

## 4. Co-Culture of *Nannochloropsis* with Microorganisms

The application of co-cultivation principles in bioprocessing has been greatly explored as the benefits of mixing cultures have been unraveled. The improvement in biomass and added-value compound productivity, enhanced growth, efficient waste byproduct removal, or facility in biomass harvest are the main advantageous traits of microalgae–microorganism co-cultivation systems. These traits are detailed further in this article.

Table 2 summarizes the cases of co-culture of *Nannochloropsis* spp. with other various microorganism species found in the literature.

### 4.1. Microalgae and Microalgae

The main characteristic of a co-culture system involving two microalgae species is the existence of competition for the same limited resources. This can trigger mechanisms of adaptation that can ultimately lead to increased biomass growth, efficient nutrient uptake, and improved lipid yields. Moreover, the complementary metabolic pathways of different species may enhance the overall production of valuable compounds, potentially leading to the discovery of new bioactive molecules with novel health-promoting properties [6]. Marine microalgae species have gained attention due to their ability to grow in saline water and with recycled nutrients, reducing the costs of freshwater. This feature makes them a cost-efficient and environmentally friendly alternative in biofuel and added-value compound production [62].

Most cases of co-cultivation of *Nannochloropsis* sp. with other marine microalgae leverage their capacity to biosynthesize compounds of interest. Maglie et al. (2021) [54] presented a comprehensive study on the co-cultivation of *N. oculata* with the marine microalga *Tisochrysis lutea*, demonstrating that while this co-cultivation strategy enhanced the production of valuable metabolites and optimized nutrient utilization, it also presented downsides. In fact, both species exhibited higher biomass growth in mono-cultures, which the authors attributed to the initial cell density used, suggesting that the inoculum ratio is also an important factor to account for when designing artificial co-cultivation systems [54]. On the other hand, co-cultivation resulted in increased chlorophyll and carotenoid content, highlighting its potential in increasing secondary metabolite productivity [54]. Regarding the lipid profile, there was a slight decrease in the total fatty acid content related, once more, to the proportion of algae in the poly-culture. Nonetheless, the combined system resulted in a richer omega-3 PUFA content and a diversified profile, with *N. oculata* contributing 17.4 mg/g DW composition in EPA and *T. lutea* providing 13.9 mg/g DW of DHA [54]. Building on this, Thurn et al. (2022) [63] optimized the combined production of EPA and DHA by co-culturing two marine oleaginous species, *T. lutea* with *M. salina*, achieving a balanced 1:1 ratio of these essential FAs after 8 days, with a reported increase in the DHA concentration of around 73% but no significant changes in EPA production improvement. The co-cultivation system enabled the simultaneous production of these omega-3 FAs, although it did not enhance the overall accumulation of EPA. Most EPA-producing organisms are unable to synthesize DHA, and vice versa, making this strategy particularly attractive in obtaining a more diverse microalgal lipid content despite the need for further optimization [63]. The authors also registered a biomass increase of 31% due to the optimal utilization of the light spectrum [63]. More recently, these authors expanded their approach to the green microalgae *N. oceanica* and *M. salina* (EPA producers) and the brown microalgae *I. galbana* (DHA producer), achieving enhanced biomass production and richer omega-3 FA profiles, likely due to the species’ capacity to absorb light at different wavelengths in the mixed culture [55]. While these studies highlight the potential of co-cultivation in producing two highly valuable bioactive compounds in a single system, they also underline important trade-offs that must be accounted for and further investigated to optimize mixed culture bioprocesses.

Several studies have reinforced the potential of poly-cultures of marine oleaginous microalgae in biofuel production, where a high PUFA content improves fuel properties and promotes carbon-neutral processes with no additional carbon being released into the atmosphere [64]. Additionally, the enrichment in omega-3 FAs in combined microalgae cultures supports their application in the nutraceutical industry, particularly in the production of alternative fish oil supplements and in the nutritional enrichment of aquaculture feed [6].

Beyond these applications, *Nannochloropsis* sp. cultivation with other microalgae species has also demonstrated increasing value in bioremediation. Davis et al. (2015) [53] explored the potential of using struvite, a phosphate mineral commonly recovered from wastewater, as a nutrient source for a combined marine culture of *M. salina* and *P. tricornutum*. The research findings showed that the nitrogen and phosphate content of struvite-based media was sufficient to support the cell culture in the same way as the standard medium, with the advantage of increasing nutrient uptake and pigment production [53]. Using a different approach, Sharma et al. (2020) [52] evaluated the bioremediation efficiency achieved when culturing microalgal consortia of both fresh (*Chlorella* sp., *Chlamydomonas reinhardtii*, *Scenedesmus bijugatus*, and *Scenedesmus dimorphus*) and seawater (*Nannochloropsis* sp.) species in sewage wastewater. One of the artificial microbial consortia resulted in the removal of ~86% of total organic carbon and 87% of chemical oxygen and reduced the nitrate and phosphate compounds by ~94%, with efficient heavy metal removal [52]. This pollutant removal capacity of microalgae co-cultures can also be applied in aquaculture to treat marine wastewater, as studied by Davis et al. (2015) [65] when designing a co-cultivation system of marine *P. tricornutum* with freshwater *Chlorella* sp. The synergistic interactions between both species resulted in a removal rate of total nitrogen of ~93% and of total phosphorous of ~96% [65]. *P. tricornutum* metabolites enhanced the photosynthetic activity of *Chlorella*, which thereby increased the bioavailability of carbon sources favorable for the heterotrophic regime of the seawater species [65]. In the same work, the authors proved the efficacy of the proposed co-culture system outdoors in actual marine wastewater, bridging the gap for application in aquaculture ponds [65]. These results demonstrate the industrial applicability of microalgae-based co-cultivation systems for WWT, as they report high nutrient removal efficiency, particularly in reducing organic carbon, nitrogen, phosphorus, and heavy metals, aligning with the requirements for large-scale bioremediation practices.

Overall, these case studies highlight the potential of marine oleaginous microalgae co-culture in bioremediation while holding increasing interest for biofuel production and dietary supplements. By improving nutrient recycling and enhancing biomass and secondary metabolite productivity, microalgae–microalgae systems offer a potentially more sustainable and cost-efficient approach.

### 4.2. Microalgae and Bacteria

The duality of microalgae–bacteria is a well-documented and naturally occurring interaction in aquatic environments. Bacteria thrive in the nutrient-rich phycosphere surrounding microalgal cells. Complex microbial consortia engage in all types of symbiotic interactions, from mutualistic nutrient cycling to QS regulation [6]. The mutualistic relationships are the focus of this section.

Mutualism between bacteria and microalgae is primarily based on nutrient exchange since microalgae work as a supplier of the dissolved organic carbon necessary for bacterial growth. Simultaneously, bacteria decompose inorganic matter, providing a myriad of nutrients, vitamin precursors (vitamin B12), growth-promoting hormones (IAA), CO_2_, and available nitrogen to microalgae [66].

The specific bacterial communities associated with microalgae can vary significantly depending on environmental factors, which influence their growth and biochemical composition in different ways. When characterizing the phycosphere of a *Nannochloropsis* sp. strain cultivated in outdoor photobioreactors, Lian et al. (2021) [33] identified eighteen bacterial strains. Among them, *Maritalea porphyrae* and *Labrenzia aggegata* were found to significantly enhance microalgae growth and chlorophyll production, whereas other bacteria, such as *Flavonacteria*, caused growth inhibition [33]. These results highlight the importance of thoroughly characterizing naturally occurring microalgae–bacterial interactions before designing a co-culture system in order to select the bacterial strains that would contribute favorably to the process.

There are several reports on the co-culture of marine microalgae with bacteria for a variety of biotechnological purposes, including WWT, biofuel production, added-value metabolite synthesis, contamination control in microalgae cultures and aquaculture ponds, and waste metabolite removal. Subasankari et al. (2020) [45] investigated the symbiotic relationship between *N. oceanica* and the halophilic bacterium *Halomonas aquamarine*. Their study revealed that bacterial co-cultivation enhanced microalgae biomass production, lipid accumulation, and carotenoid synthesis. This improvement was attributed to the production of siderophores and vitamins by *H. aquamarine*, which function as algal growth factors, leading to a 14% increase in lipid content [45]. Similar beneficial effects were observed in co-cultures involving other marine oleaginous microalgae, such as *I. galbana* and *P. tricornutum*, with bacterial strains from the *Marinobacter* genus. These mixed cultures not only enhanced biomass productivity but also increased lipid accumulation, with *I. galbana* yielding higher concentrations of DHA [67,68]. These findings underscore the potential of bacterial co-cultivation strategies to enhance the production of bioactive compounds with potentially significant health benefits.

One of the bottlenecks in large-scale algal biotechnology is biomass harvesting, which often requires high-energy-consuming techniques [61]. This cost-driver step is particularly relevant in biofuel production for the dewatering of cells [69]. To address this, researchers have explored bacterial co-cultivation as a strategy to enhance microalgal aggregation and reduce energy requirements during harvesting processes. Powell and Hill (2013) [58] exploited the role of *Bacillus* sp. bacteria in the aggregation of *N. oceanica* cultures. Their results showed that cell accumulation rapidly occurred in response to the bacterial secretion of extracellular polymeric substances (EPSs), which promoted cell adhesion and clustering [58]. Similarly, Tran et al. (2020) [56] identified bacterial isolates from the *Rhodobacterales*, *Flavobacteriales*, and *Sphingomonadales* orders as key players in promoting EPS production and the consequent aggregation of *N. oceanica*, leading to improved biomass recovery. These findings support bacterial co-cultivation strategies as a cost-effective and sustainable approach for mitigating harvesting challenges.

In addition to aggregation, another major limitation in microalgal bioprocessing is the difficulty in degrading the stable and resistant cellulose-rich cell wall to gain access to the intracellular metabolites. As previously stated, *Nannochloropsis* species, in particular, are known for their thick and rigid walls, which constitute an obstacle for intracellular lipid extraction [19]. Generally, mechanical or chemical pre-treatment steps are employed to break down or weaken cell walls; however, these methods have the downside of being costly and aggressive, with the possibility of causing degradation of the target products. Co-culture can be applied as a more sustainable alternative, as specific microbial strains might help overcome cell wall resistance, improving the bioavailability of intracellular compounds for food and biotechnological applications. Understanding the cell wall composition is crucial to select a co-cultivation partner that could hydrolyze the cell wall components without compromising the intracellular products. Muñoz et al. (2014) [59] studied the enzymatic capacity of bacteria to hydrolyze microalgal cell walls by co-culturing microalgae with several species of bacteria. Their results showed that the most efficient strain in *N. gaditana* cell wall degradation was *Raoultella ornithinolytica*, whereas it proved to be ineffective towards *Botryococcus braunii* [59]. This disparity of results was proposed to be caused by the influence of external factors on cellulolytic bacterial metabolic performances, highlighting the need for a deeper understanding of interspecies relationships [59]. This system promoted bacterial enzymatic cell wall degradation, enhancing lipid recovery without the need for expensive and energy-consuming pre-treatment processes [59]. The increase in the bioavailability of lipids represents, once more, an advantageous trait in the biofuel production, although it could not be applied in the food sector, as *R. ornithinolytica* is known to cause infections in humans [70]. While their case study presents the potential of microalgal–bacterial interactions as an alternative to costly pre-treatment processes, further research should prioritize identifying non-pathogenic species.

In conclusion, bacterial co-cultivation offers the opportunity to enhance *Nannochloropsis* sp. productivity and lipid accumulation and facilitate biomass harvesting and lipid extraction, being a promising and economically efficient approach for the scale-up of algal biotechnology.

### 4.3. Microalgae and Fungi

Microalgae–fungi associations are naturally occurring symbiotic associations that can improve biomass growth, lipid accumulation, and WWT efficiency. The interest in the co-culture of these microorganisms focuses on the self-pelletization capacity of filamentous fungi, which enables them to aggregate with each other into fungal pellets [61]. When in association, fungi work as bio-flocculant agents, entrapping microalgae in their pellets, thereby facilitating the separation of biomass from the liquid fraction [61]. In accordance with the previously identified bottlenecks of microalgal bioprocesses, this bio-flocculation property is of great significance in industrial applications, as it simplifies and cuts the costs of cell harvesting. Beyond cell aggregation, microalgae and fungi co-cultures can enhance the lipid production of TAGs and PUFAs, both of which are valuable for biofuel production and nutraceutical applications. Furthermore, among all microalgae–microbial associations, algae–fungi systems have demonstrated the highest efficiency in WWT due to the combined action of microalgal nutrient assimilation with fungal organic matter degradation [25].

Du et al. (2018) [60] explored the co-culture of *N. oceanica* with the oleaginous fungus *Mortierella elongata*, aiming for bio-flocculation and lipid yield improvement. The study implemented a cultivation strategy in which the microorganisms were grown separately, allowing *M. elongata* to develop its mycelial network before being added to the mixed culture. The results exhibited a significant enhancement in biomass aggregation, with the fungus species efficiently capturing microalgal cells in its network and thereby facilitating cell separation and lipid extraction. Moreover, the co-culture led to an increase in lipid accumulation in both oleaginous species, with a significant rise in TAG and PUFA contents. Notably, *M. elongata* also enhanced the production of DHA due to the high salinity of the medium. The study also applied genetic engineering techniques to further optimize the FA profile of *N. oceanica* [60]. A similar approach was explored by Wrede et al. (2014) [61] by co-cultivating the filamentous fungus *Aspergillus fumigatus* with various marine oleaginous microalgae species (*D. salina*, *N. oculata*, and *T. chuii*). The results confirmed that fungal–microalgal interactions improved bio-flocculation up to 90% after 24h of co-cultivation for almost all the tested microalgal species. Fungal mycelia trapped microalgal cells within their filaments, simplifying biomass harvesting. In addition, the co-culture increased lipid yields and diverse content in TAGs and PUFAs [61]. Beyond biofuel production, their study proved the efficacy of combined cultures of *A. fumigatus* with microalgae in WWT. The fungi successfully degraded organic matter while the microalgae assimilated the resultant nitrogen and phosphorous, creating an efficient circular system with potential for large-scale wastewater bioremediation [61].

The Integration of microalgae and fungi represents a promising, scalable, and sustainable strategy for addressing several challenges in biofuel production, microalgal harvesting, and environmental remediation. This co-culture pairing holds the potential for several industrial applications if the right strains and conditions are applied.

#### Microalgae and Yeasts

Similar to other microbial interactions, the co-cultivation of microalgae and yeasts has been highlighted in previous studies, demonstrating its biotechnological potential, particularly in enhancing lipid production [71]. This approach has been explored for applications in biofuel production, aquaculture feed, WWT, and added-value compounds synthesis [72]. Microalgae and yeast form a complementary metabolic relationship, where photosynthetic microalgae produce O_2_ and organic carbon sources that support yeast growth, while yeasts produce CO_2_ and nutrients that stimulate microalgal metabolism [73]. This exchange of gas and metabolites not only optimizes biomass growth but also improves lipid accumulation, as yeasts can increase the bioavailability of key metabolites required for microalgae lipid biosynthesis [6]. Additionally, the simple cultivation requirements of yeasts and their ability to thrive on diverse feedstocks allow for the reuse of industrial or agricultural waste streams, making these co-culture systems useful in applications associated with WWT [74,75]. Another characteristic of yeasts is their capacity to degrade heavy metals by bioaugmentation, making them valuable in the environmental treatment of contaminated sites [76].

While co-cultivation strategies have been explored in freshwater microalgae–yeast systems, research on marine oleaginous microalgae, particularly for *Nannochloropsis* sp., remains limited. However, Cai et al. (2007) [77] analyzed the growth and biochemical composition of a mixed culture of the marine microalgae *I. galbana* and the yeast *Ambrosiozyma cicatricose*, showing that when cultured together, both organisms reached higher growth rates and increased FA production. However, the overall lipid concentrations did not significantly surpass those of the mono-cultures, demonstrating that despite the potential of these systems, there is space to optimize their interactions [77].

Given the high lipid productivity of certain marine microalgae and the metabolic versatility and efficiency of yeasts, further research into optimizing strain selection and culture conditions could unlock new opportunities for scalable and cost-effective alternatives for biotechnological applications [72]. The integration of yeasts in marine microalgal cultures presents an avenue of research with economic and sustainable potential and an opportunity for further scientific investigation.

### 4.4. Co-Culture of Other Microalgae with Microorganisms

The literature on microalgae–microorganism consortia is extensive, particularly regarding biotechnological purposes. Several reviews have been published on microalgal co-cultivation strategies for biofuel production [78], bioremediation and WWT [6,79], and the synthesis of bioactive compounds [25,80]. Among the microalgae studied in poly-cultures, freshwater *Chlorella* spp. and *Scenedesmus obliquus* stand out as some of the most common [81]. *Chlorella* spp. are particularly valued for their secondary metabolite production, robust growth with diverse substrates, and compatibility with various microorganisms, while *S. obliquus* is recognized for its high lipid content and efficient nutrient uptake [81]. However, the co-cultivation of these and other microalgae species falls outside the scope of this review, which is primarily centered on *Nannochloropsis* spp. Given their classification as marine oleaginous microalgae, it is relevant to compare their co-cultivation studies with those involving other microalgae from the same ecological and metabolic niche (presented in Table 1). Table 3 provides a summary of co-culture studies conducted with marine oleaginous species besides *Nannochloropsis*, offering a broader understanding of co-cultivation strategies in this specific group.

Although the category of marine oleaginous species includes more species (Table 1), the ones presented in Table 3 are the ones that were found in published articles on co-culture. In broad lines, *Phaeodactylum tricornutum* is a marine diatom widely recognized for its ability to synthesize significant amounts of EPA and fucoxanthin [9,10]. In co-culture systems, *P. tricornutum* has been paired with other microalgae or bacterial partners (e.g., *Marinobacter* sp.) to enhance lipid and biomass productivity and optimize wastewater treatment [68,82]. In comparison with *Nannochloropsis*, *P. tricornutum* can be more sensitive to fluctuations in temperature and light variations, besides requiring complex and expensive harvesting steps in the downstream processing, which constitutes a challenge at the industrial scale [94].

*Isochrysis galbana*, valuable for its high PUFA content, containing both EPA and DHA, is usually applied in aquaculture feed and nutraceutical formulations [11,12]. Co-culture systems involving *I. galbana* and bacterial strains (*Alteromonas* sp., *Labrenzia* sp.) have shown improvements in biomass yield, lipid profile, and even pathogen inhibition [88]. However, *I. galbana* generally produces lower overall lipid yields compared to *Nannochloropsis* (Table 1).

Species from the *Tetraselmis* genus are known for their ability to adapt to various salinity levels besides their rentable lipid accumulation [13,14]. Co-culture studies often focus on the microalgae’s capacity to induce flocculation, promoting the harvesting step in other microalgae cultures [89]. While *Tetraselmis* sp. demonstrates strong resilience and decent growth rates, its lipid profile tends to be less dominated by polyunsaturated fatty acids (PUFAs) than that of *Nannochloropsis*. Consequently, *Nannochloropsis* remains a more attractive option for industries seeking high-value PUFAs.

Finally, *Dunaliella salina* is best known for its high β-carotene content, making it a valuable asset in the nutraceutical and cosmetic sectors [15]. The few studies found on co-culture approaches paired *D. salina* with bacterial strains (*Halomonas* sp.), aiming for enhanced biomass production and carotenoid yield while simultaneously addressing bioremediation challenges [93]. Although *D. salina* can accumulate lipids under stress, it is primarily cultivated for carotenoids rather than PUFAs, unlike *Nannochloropsis*.

While each marine oleaginous microalga has its unique advantages and added-value metabolites, *Nannochloropsis* excels in producing EPA-rich lipids, often exhibiting robust growth, high biomass, and PUFA yields and significant tolerance to salinity and temperature changes. This combination of resilience and high-value lipid production makes *Nannochloropsis* especially appealing for industrial applications in the food, feed, biofuel, and bioremediation sectors, with more published research on co-culture approaches than the other marine oleaginous microalgae. Consequently, *Nannochloropsis* remains a prime candidate for large-scale co-cultivation systems designed to optimize PUFA production while maintaining cost-effectiveness. Nonetheless, further exploration of other valuable marine species presents an opportunity to uncover novel traits and applications that could enhance the existing bioprocesses.

## 5. *Nannochloropsis* sp. in Food and Feed

### 5.1. Microalgae in Food and Feed

In this review, as in previous reviews, microalgae consortia are presented as a valuable strategy to optimize biofuel production, WWT, bioremediation, and added-value metabolite production. The ability to engineer symbiotic relationships between microalgae and microorganisms has led to scalable and cost-effective solutions for some of the major bottlenecks in microalgal biotechnology, including enhanced biomass harvesting, improved lipid productivity, and efficient nutrient recycling [6]. However, the full potential of using these microorganisms in the food sector is still a topic to be explored. On the other hand, from a nutritional point of view, it has been stated that microalgae are a promising asset for food and feed purposes due to their high content in proteins, carbohydrates, lipids and carotenoids, holding great potential in representing a natural, sustainable, and healthy alternative to other protein sources and dietary supplement formulations.

In livestock, incorporating microalgal biomass in feed formulations has been shown to enhance animal growth, improve their immune response, and increase their resistance towards bacterial and viral infections [95]. In aquaculture, microalgae serve as sustainable alternatives to traditional fishmeal (i.e., fish farming feed composed of smaller fish or fish waste), contributing to releasing the pressure on wild fish stocks. Whether as an exclusive feed compound or as part of regular feed, microalgae have been shown to improve the nutrient content, color, and overall health of aquaculture mollusks, crustaceans, shrimps, and fish [96]. Moreover, terrestrial animal feeds supplemented with microalgae have demonstrated improvements in meat quality caused by the antioxidant properties and the high content of PUFAs and pigments in microalgae [96].

In human consumption, the introduction of microalgae in the human diet is becoming more popular by the day, whether in the form of tablets, dried powders, omega-3-rich oil capsules, proteins, carotenoids, or other food supplement formulations. Moreover, as they can be easily incorporated in a variety of food products, their versatility has facilitated their consumption as ingredients in common foods such as bread, snacks, dairy products, and beverages. However, the value of microalgae in cooking goes beyond their nutritious content since they are also beneficial for their organoleptic, rheological, and shelf-life properties [95]. As a viable alternative to conventional fish oil supplements and animal protein, microalgae have the potential to help reduce the pressure on traditional food systems while still ensuring global food security.

The co-cultivation of microalgae with other microorganisms also presents interesting opportunities in the food and feed industry, particularly in enhancing nutritional quality while aiming for more sustainable processes. Combined cultures of microalgae are used in aquaculture systems to serve as rich nutritional feed and probiotics or to mitigate potential diseases and ensure overall seafood health, contributing to more sustainable aquaculture practices [97]. In the same way, these principles could be applied to human nutrition. The literature on microalgal poly-cultures designed for human consumption is still scarce. Most commercialized products combine multiple microalgal biomasses or their bioactive products in one nutrient-rich supplement [98]. Tyus (2016) [98] explored this idea by analyzing the protein, carbohydrate, and lipid production of microalgae co-cultures so that they could be applied in nutraceuticals. The author concluded that the biochemical value of the co-culture system resulted in higher lipid, carbohydrate, and protein profiles and mono-culture biomass, highlighting the potential behind exploring microalgal mixed cultures in human supplement formulations. More studies on the matter should be performed to investigate the potential of microalgae–microalgae and microalgae–microbial systems in the development of functional foods, as it has been displayed throughout this work that it can be highly advantageous in the nutraceutical industry.

### 5.2. The Potential of Nannochloropsis in the Alternative Protein World

The growing demand for sustainable, safe, and animal-free protein sources has accelerated research on finding alternative sources of proteins, with microalgae emerging as promising alternatives to animal protein. Their rapid growth and high protein content, composed of all essential and non-essential amino acids, have attracted significant attention for their use in plant-based food [99].

*Nannochloropsis* sp., in particular, has a high protein content of around 30% of its DW, with a balanced ratio between essential and non-essential amino acids, making it a viable protein source in food formulations and a sustainable alternative to animal proteins [100]. Moreover, due to its ability to rapidly accumulate omega-3 FAs, essential for human health, *Nannochloropsis* sp. holds great potential for food application as it can provide a rich nutritional value to microalgae-based products [24].

Beyond the simple use of *Nannochloropsis* biomass or bioactive compounds, the application of this marine species in food can be further enhanced by resorting to co-cultivation principles. As it was stated in the previous section, the synergistic interactions between *Nannochloropsis* and complementary microorganisms not only enhance biomass productivity and nutrient recycling but also stimulate the production of bioactive metabolites—such as antioxidants, vitamins, and flavor compounds [54]. These approaches can be used to improve the sustainability and the nutritional and sensory qualities of microalgae food products. Moreover, the incorporation of co-culture systems in food technology can bring further advantages in overcoming *Nannochloropsis* biotechnological hurdles, such as lower digestibility and limited bioavailability of intracellular compounds [59]. This represents a gap in the scientific research on *Nannochloropsis* and an opportunity for further investigation. Co-culture systems offer a more robust nutritional profile, reduced production costs, and improved sustainability, making them an attractive line of research for developing high-quality, functional, plant-based foods and alternative protein sources.

The usage of microalgae can also be expanded to the novel field of cellular agriculture due to their nutritional benefits and suitability for co-culture. Innovative research is beginning to explore the co-cultivation of microalgae with animal cells to create integrated systems that could enhance the nutritional profile of cultured meat and seafood [101]. Like the previously described microbial interactions, microalgae would provide the essential nutrients, vitamins, and growth factors that support animal cell proliferation while simultaneously recycling metabolic waste products, such as CO_2_ and nitrogen and phosphorous compounds [101]. Besides nutritional enhancement, a co-culture system would also reduce the costs associated with cultured food and contribute to closed-loop production with lower environmental impacts [102]. As a matter of fact, studies have shown that microalgae can successfully be integrated as part of the cultured meat production process in various ways: (i) microalgae extracts can be employed as a nutrient source, replacing the need to use Fetal Bovine Serum or basal medium [103], (ii) recyclers of waste products in the spent culture medium [104], and (iii) incorporated in scaffolds to promote thicker and healthier tissues by providing a constant flux of O_2_ and nutrients to the animal cells in co-culture [105].

In the same way, microalgae from the *Nannochloropsis* genus have the potential to be introduced into cultivated products, mainly in cultivated seafood production. Their high content of PUFAs would not only contribute to the nutritional enhancement in omega-3 FAs, especially EPA, but also potentially provide a fish flavor and smell to the cultivated product, approximating the cultivated seafood to conventional products [106].

Although no research on direct co-culture systems between marine oleaginous microalgae and animal cells was found, the promising synergy observed in microalgae–microbial systems provides a strong foundation for further exploration. *Nannochloropsis* sp. constitutes a versatile resource for the development of alternative food products, offering both nutritional enrichment and process sustainability in emerging cellular agriculture technologies by taking full advantage of its marine bioactive compounds.

### 5.3. Toxicology, Safety, and Regulatory Aspects of Nannochloropsis *sp*.

The growing interest in *Nannochloropsis* sp. is derived from its rich chemical composition, particularly in PUFAs, as it is a sustainable and vegetarian source of omega-3 FAs for both feed and food applications. Nonetheless, one of the setbacks related to the integration of microalgae biomass in food is ensuring its safety for humans and animals. So far, no toxins produced by the *Nannochloropsis* genus are known [19].

A significant setback reported regarding the use of *Nannochloropsis* in food and feed is related to its lower digestibility, primarily due to its thick cell walls [24]. To overcome this downside, cell wall disruption strategies are required. Traditional physical or chemical pre-treatment methods can be costly and energy-consuming [59]. Co-culture strategies offer a more sustainable alternative by utilizing bacterial or fungal pre-treatments to degrade the *Nannochloropsis* cell wall [59]. To accomplish this, it is important to understand the precise composition and structure of the *Nannochloropsis* cell wall to select optimal co-culture partners that could hydrolyze the highly resistant algaenan outer layer while also aiming for non-pathogenic strains [22]. Further investigation should be performed on the potential of opting for co-cultivation strategies as sustainable and cost-effective alternatives to improve the bioavailability and digestibility of *Nannochloropsis*.

Additionally, there is a great concern related to the capacity of microalgae and *Nannochloropsis* spp. to bioaccumulate contaminant substances intracellularly, such as heavy metals, polychlorinated biphenyls, and toxins present in the surrounding environment [19,107]. To date, there are no reports of allergic reactions directly attributed to *Nannochloropsis* sp., although its cultivation must be carefully managed under controlled and clean conditions to minimize risks of contamination. In fact, the major legislation constraints are precisely associated with the safe use of microalgae in food, emphasizing the necessity to thoroughly study the toxicity profile of microalgae-based products before introducing them in the human diet [108].

In summary, this microalga, by itself, does not constitute a toxic hazard for human consumption, animals, or plants. However, more rigorous toxicological studies should be performed. A report published by the European Union (EU) Joint Research Centre classified *Nannochloropsis* as a safe source of EPA for food supplements in the human diet (with no production of toxins), provided that the quality control regulations are respected [109]. In the EU market, microalgae fall into the Novel Food Regulation No. 2015/2283, which has approved 20% of the 150 regularly consumed algae species [108].

While established species, such as *Arthrospira plattensis* (commonly known as Spirulina), *Chlorella*, and *Aphanizomenon flos-aquae*, are considered Not Novel Foods since they have been largely consumed prior to 1997, more recently consumed species, such as *Odontella aurita*, *Ulkenia* sp., *Tetraselmis chuii*, *Haematococcus pluvialis* and *Schizochytrium* sp., have been approved and categorized as Novel Foods [110,111].

*Nannochloropsis* sp. is currently on the list of microalgae species to be approved for food and food supplements in the EU. As a source of valuable bioactive compounds, such as EPA, lipids, and arachidonic acid, this species has drawn attention for its valuable use in food. The EU approval of microalgae species for human consumption is always evolving and does not necessarily accompany the worldwide legislation. In fact, some microalgae and microalgae byproducts, despite being produced in Europe, are commercialized elsewhere [110]. In fact, there are already *Nannochloropsis*-based products being commercialized outside Europe. For example, Qualitas Health produces liquid capsules rich in EPA oil secreted by *N. oculate* and Optimally Organics produces a nutritional supplement composed of *N. gaditana* dried powder, laying the grounds for the development of future products [112].

*Nannochloropsis* sp. inclusion in the EU’s Novel Foods list is progressing, with ongoing research and regulatory developments paving the way for its approval. The process is being facilitated as some products of *Nannochloropsis* species, such as *N. gaditana* or *N. oculata*, go through the acceptance process, see approval, and are commercialized in specific countries [24].

## 6. Current Challenges and Future Perspectives

The co-cultivation of marine oleaginous microalgae, particularly of the *Nannochloropsis* genus, with other microorganisms presents a promising approach for enhancing biomass production, nutrient recycling, and high-value metabolite production, which supports their versatile applications in food, feed, nutraceuticals, biofuel, WWT, and bioremediation.

However, several technical, research, and scalability challenges must be addressed to take advantage of the full potential of co-culture systems. In fact, scalability has become one of the biggest challenges associated with microalgae-based co-culture systems. Growth rate disparity, nutrient competition, and variable environmental conditions can lead to the dominance of one species over the other, reducing the overall system efficiency [6]. Moreover, a thorough study on the strains utilized in the co-culture system must be performed first to ensure that there is a beneficial symbiotic relationship that would generate a positive outcome while opting for strains with the potential for large-scale cultivation. Additionally, contamination control also poses a significant challenge, which is fundamental to maintaining selective growth conditions without inhibiting beneficial interactions or causing safety concerns, especially in the production of functional foods [25].

In that sense, further research is needed to identify compatible microbial partners that can enhance the productivity and stability of marine microalgae co-cultures, ensuring that non-pathogenic microorganisms are utilized in food and nutraceutical bioprocessing. Advances in genetic engineering and synthetic biology offer potential solutions for improving compatibility, optimizing metabolic rates, and biochemical synthesis. Investigating novel microbial consortia could further expand the range of beneficial interactions and applications. Furthermore, appropriate co-cultivation systems should be designed to better potentiate the simultaneous growth of both species by regulating nutrient availability and light exposure and ensuring the overall ideal culture conditions that are essential for optimizing co-culture performance [26].

Despite these challenges, the future of marine oleaginous microalgae co-cultivation is promising and far from being unraveled. The role of these photosynthetic organisms in microbial consortia is already advancing, with several case studies supporting the advantages they bring to numerous biotechnological applications. The ability to enhance the nutritional profile and bioactive compounds in co-cultures makes them a valuable resource for the development of plant-based foods and dietary supplements of marine origin; therefore, research on this theme is highly encouraged. Co-culture approaches should be further investigated as sustainable alternatives to overcome some of the challenges related to *Nannochloropsis* bioprocessing, such as low digestibility and harvesting inefficiency while enhancing added-value metabolite production.

Developing innovative co-cultivation strategies could unlock new ways to enhance the bioavailability, functionality, and sensory attributes of *Nannochloropsis*-based food products, ultimately facilitating their integration into the alternative protein market. The potential co-culture of these microalgae with animal cells is still at its early stages, constituting a new avenue of research and holding great potential for future applications in food and cellular agriculture.

## 7. Conclusions

In this review, it was demonstrated that *Nannochloropsis* sp., with its high nutritional value, adaptability, and simple cultivation requirements, holds great promise as a versatile co-cultivation partner for various biotechnological applications. Its potential to enhance biomass productivity, nutrient recycling, and bioactive compound production in poly-cultures makes it a valuable candidate for industries ranging from food and nutraceuticals to biofuels and wastewater treatment. The value of marine bioactive compounds in human health was detailed, as the role of these valuable microorganisms in human and animal diets is of significant interest, highlighting the need to explore novel strategies for introducing marine microalgae co-culture in food technology. However, to fully unlock these opportunities, further research is required to overcome technical challenges, such as optimizing species compatibility, balancing nutrient dynamics, and scaling up co-culture systems. Addressing these hurdles through innovative co-culture approaches will be crucial in paving the way for the sustainable and commercially viable application of *Nannochloropsis* in various sectors. Expanding research efforts in this field will not only improve the efficiency and feasibility of these systems but also contribute to the broader goal of developing more sustainable biotechnological solutions across various industries, particularly in food technology.

## Figures and Tables

**Table 1:** Marine oleaginous microalgae species with biotechnological interest and their valuable bioactive compounds, total lipid content under optimal conditions, and applications.

Marine Microalgae Species	Bioactive Compounds	Total Lipid Content	Applications	Reference
*Nannochloropsis* spp.	PUFAs, EPA	30–60%	Biofuel, nutraceuticals, feed, food	[7,8]
*Phaeodactylum* *tricornutum*	PUFAs, EPA, fucoxanthin	20–30%	Nutraceuticals, pharmaceuticals, biofuel, cosmetics	[9,10]
*Isochrysis galbana*	PUFAs, DHA, EPA	25–30%	Aquaculture feed, nutraceuticals	[11,12]
*Tetraselmis* spp.	PUFAs	12–15%	Aquaculture feeds, biofuel, wastewater treatment	[13,14]
*Dunaliella salina*	PUFAs, Beta-carotene, lutein	7%	Biofuel, cosmetics, nutraceuticals, pharmaceuticals	[15]
*Schizochytrium* spp.	PUFAs, DHA	(up to 60% under stress)	DHA-rich oils, oral vaccines	[16,17]
*Pavlova lutheri*	PUFAs, DHA, EPA	50–70%	Aquaculture feed, biofuel	[18]

**Table 2:** Published literature on the co-culture of *Nannochoropsis* sp. with other microorganisms and respective applications.

Co-Culture Type	Microalgae Species	Co-Culture Species	Application	Reference
Microalgae– microalgae	*Nannochloropsis* sp.	MAC1 ^1^: *Chlorella* sp., *Chlamydomonas reinhardtii*, *Scenedesmus bijugatus*, and *Oscillatoria* MAC2: *Chlorella* sp., *Kirchnella*, *Scenedesmus dimorphus*, and *Microcoleus*	- Sewage wastewater treatment. - Heavy metal removal by bioaugmentation.	[52]
	*M. salina*	*Phaeodactylum* *tricornutum*	- Wastewater treatment.	[53]
	*N. oculata*	*Tisochrysis lutea*	- Biomass growth and lipid production.	[54]
	*N. oceanica*	*Isochrysis galbana*	- Biomass growth and lipid production.	[55]
Microalgae– bacteria	*N. oceanica*	*Halomonas aquamarina*	- Biomass growth and lipid production.	[45]
	*N. oceanica*	Bacterial isolates from the Rhodobacterales, Flavobacteriales, and Sphingomonadale orders	- Facilitate biomass aggregation.	[56]
	*N. oceanica*	Genera *Algoriphagus, Oceanicaulis*, and *Marinobacter*	- Enhance productivity and stability of cell cultures.	[57]
	*N. oceanica*	*Bacillus* sp.	- Cell aggregation for biofuel production.	[58]
	*M. gaditana*	*Raoultella ornithinolytica*	- Cell wall degradation for biofuel production.	[59]
Microalgae–fungi	*N. oceanica*	*Mortierella elongata*	- Biofuel production. - Harvesting efficiency.	[60,61]
	*N. oculata*	*Aspergillus fumigatus*	- Wastewater treatment. - Lipid production. - Harvesting efficiency.	[61]

^1^ MAC—microalgae consortia.

**Table 3:** Published studies on the co-culture of various marine oleaginous microalgae species with other microorganisms and respective biotechnological applications.

Marine Microalgae Species	Co-Culture Type	Co-Culture Partners	Application	Reference
*Phaeodactylum* *tricornutum*	Microalgae– microalgae	*Chlorella* sp.	- Marine aquaculture wastewater treatment.	[65]
		*Dunaliella salina*	- Biomass growth and lipid and chlorophyll production.	[82]
		*Aurantiochytrium limacinum*	- EPA and DHA production.	[83]
	Microalgae–bacteria	*Marinobacter* sp.	- Biomass growth and lipid production.	[68]
		*Thalassospira* sp.	- Bisphenol removal from media.	[84]
		*Stappia* sp.	- Biomass growth and lipid and carotenoid production.	[85]
*Isochrysis* *galbana*	Microalgae– microalgae	*Chaetoceros calcitrans*	- Added-value metabolite production.	[86]
	Microalgae–bacteria	*Thalassiosira pseudonana*	- Fishery wastewater treatment.	[87]
		*Marinobacter* sp.	- Biomass growth and DHA production.	[67]
		*Alteromonas* sp.	- Biomass growth and metabolite production.	[88]
		*Labrenzia* sp.	- Biomass growth and metabolite production.	[88]
	Microalgae–yeast	*Ambrosiozyma cicatricosa*	- Biomass growth.	[77]
*Tetraselmis* spp.	Microalgae– microalgae	*T. lutea* and *Microchloropsis salina*	- EPA and DHA production.	[63]
		*T. sueccia*, and *Chlorella* sp., *Nannochloropsis* sp.	- Bio-flocculation for cell harvesting.	[89]
	Microalgae–bacteria	*T. striata* and *Pelagibaca* *bermudensis*, *Stappia* sp.	- Biomass growth and lipid production.	[90]
		*T. chuii*, and *Muricauda* sp.	- Biomass growth.	[91]
	Microalgae–fungi	*T. suecica* and *Aspergillus* *fumigatus*	- Bio-flocculation for cell harvesting, biomass growth, and lipid production,	[92]
*Dunaliella* *salina*	Microalgae–bacteria	*Halomonas mongoliensis*	- Bisphenol removal from wastewater.	[93]

## Data Availability

Not applicable.

## Abbreviations

The following abbreviations are used in this manuscript:

DHA	docosahexaenoic acid
DW	dry weight
EPA	eicosapentaenoic acid
EPS	extracellular polymeric substance
EU	European Union
FA	fatty acid
IAA	indole-3-acetic acid
MUFA	monounsaturated fatty acid
PUFA	polyunsaturated fatty acid
SFA	saturated fatty acid
TAG	triacylglycerol
QS	quorum sensing
WWT	wastewater treatment
